# Insight into the Contribution and Disruption of Host Processes during HDV Replication

**DOI:** 10.3390/v11010021

**Published:** 2018-12-31

**Authors:** Gabrielle Goodrum, Martin Pelchat

**Affiliations:** Department of Biochemistry, Microbiology and Immunology, Faculty of Medicine, University of Ottawa, Ottawa, ON K1H 8M5, Canada; gabrielle.goodrum@gmail.com

**Keywords:** hepatitis delta virus, host–pathogen interaction, viral disruption of host processes

## Abstract

Hepatitis delta virus (HDV) is unique among animal viruses. HDV is a satellite virus of the hepatitis B virus (HBV), however it shares no sequence similarity with its helper virus and replicates independently in infected cells. HDV is the smallest human pathogenic RNA virus and shares numerous characteristics with viroids. Like viroids, HDV has a circular RNA genome which adopts a rod-like secondary structure, possesses ribozyme domains, replicates in the nucleus of infected cells by redirecting host DNA-dependent RNA polymerases (RNAP), and relies heavily on host proteins for its replication due to its small size and limited protein coding capacity. These similarities suggest an evolutionary relationship between HDV and viroids, and information on HDV could allow a better understanding of viroids and might globally help understanding the pathogenesis and molecular biology of these subviral RNAs. In this review, we discuss the host involvement in HDV replication and its implication for HDV pathogenesis.

## 1. Introduction

Hepatitis delta virus (HDV) was discovered in 1977 following the identification of a new antigen present exclusively in hepatocytes from a patient having the hepatitis B virus (HBV) surface antigen (HBsAg) [1]. Originally considered a variant of the HBV nucleocapsid, a subsequent study showed a new hepatitis agent distinct from HBV. The detected antigen was termed the delta antigen (HDAg or δ) and later associated with a novel virus termed HDV. This unique pathogen has its own genus called the deltavirus and, to this day, is classified into eight major clades with sequence divergence of as much as 40% associated with its geographic origin [2]. HDV causes one of the most serious and rapidly progressive viral hepatitis, and is associated with a greater risk of developing fulminant hepatitis, hepatocellular carcinoma, cirrhosis, and liver failure [3]. Because alpha-interferon and current antiviral agents have little effect long term on HDV, liver transplantation is the only viable therapeutic option for the treatment of end-stage HDV-related liver disease [4].

HDV is a defective and satellite virus of HBV, requiring HBV envelope proteins (HBsAg) for its propagation (reviewed in Reference [5]). However, HDV shares no sequence similarity with its helper virus and replicates independently. Like HBV, HDV transmission occurs via contact with infectious body fluid, which implicates sexual transmission, the use of contaminated needles, and transmission via the parenteral route [6,7,8]. HDV infection has been reported worldwide but is predominantly found in countries without easy access to HBV vaccination and/or to education on the risk of infectious disease transmission. Since the implementation of HBV vaccination in 1980, the HDV prevalence was reduced considerably [9]. However, the prevalence of HDV is still high in injection drug users, especially for those infected with the human immunodeficiency virus [10]. It is estimated that approximately 15 to 20 million people are co-infected or superinfected with HDV, which represents roughly 5% of people affected with HBV worldwide [11]. However, immigration from areas where HDV remains endemic constantly reintroduces the infection, and several countries do not report or do not systematically test for HDAg specific antibodies in HBV infected patients, causing an incomplete global estimation of the number of people affected [3].

## 2. The Genome of HDV and Its Replication

**Figure 1:**
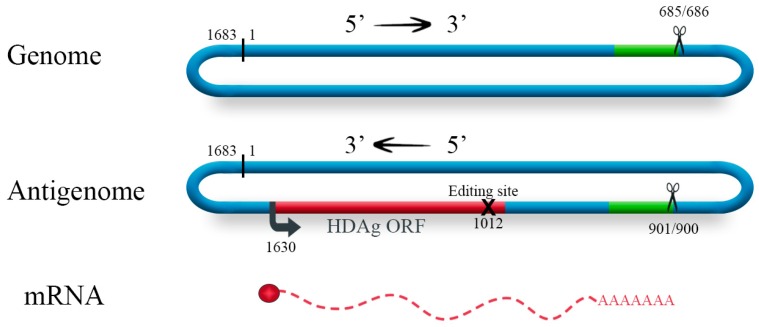
Hepatitis delta virus genomic RNA, antigenomic RNA and mRNA. The delta ribozymes are represented by the green boxes on both strands and the cleavage sites are indicated by scissor symbols. The delta antigen (HDAg) open reading frame (ORF) is represented in the antigenome by the red box, the arrow on position 1630 indicates its transcription start site and the X in the ORF represents the ADAR-1 editing site at a location corresponding to the termination codon of HDAg-S.

HDV is the smallest known human RNA virus. Its genome consists of a small (~1700 nucleotides) single-stranded, circular RNA molecule and folds into an unbranched, rod-like structure due to 74% self-complementarity (Figure 1) [5]. It includes two complementary self-cleaving motifs (i.e., delta ribozymes) and a single open reading frame encoding two viral proteins: the small and the large delta antigens (HDAg-S and HDAg-L). These two proteins are mostly identical in sequence except that HDAg-L (214 amino acids) contains 19–20 additional amino acids at its C-terminus resulting from RNA editing of antigenomic HDV RNA at a location corresponding to the termination codon of HDAg-S (195 amino acids) gene by a host adenosine deaminase that acts on RNA (ADAR-1) [12,13]. Despite being mostly identical in sequence, these two proteins have distinct functions: HDAg-S is essential for HDV accumulation, while HDAg-L is necessary for virion assembly [5]. However, the precise mechanism of action of the two proteins is still largely unknown.

**Figure 2:**
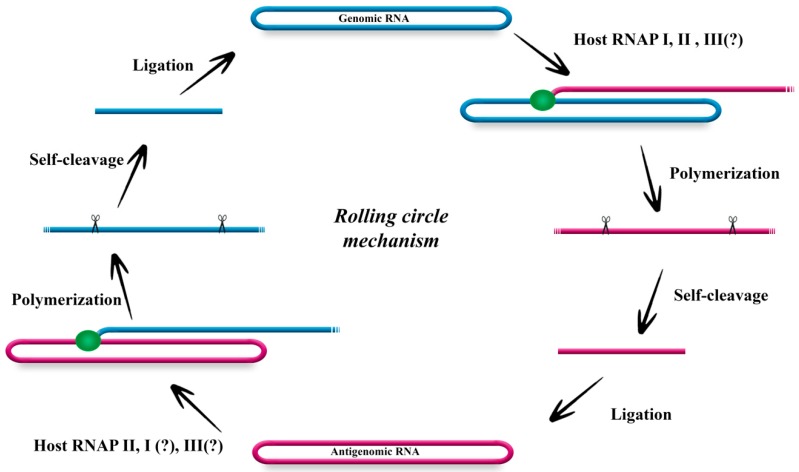
Hepatitis delta virus (HDV) replication model: symmetrical rolling circle. HDV genomic strand (blue) is used as a template to generate the antigenomic RNA using a host polymerase to generate a multimeric linear strand. This strand is self-cleaved by endogenous delta ribozymes (scissor symbols) and presumably ligated by a host enzyme to form the antigenomic monomeric strand (pink). The same steps are repeated on the antigenomic strands to produce a newly synthesized genomic strand. The genomic template is also used for the antigen mRNA synthesis. Question marks next to several RNAPs indicate that the involvement in HDV biology remains controversial.

Even though it requires the HBV envelope proteins for virion production, HDV replicates independently of HBV and relies entirely on its host for replication. HDV replicates via a symmetrical, rolling circle mechanism (Figure 2). Replication of the infectious circular monomer of genomic polarity produces linear, multimeric strands, which are cleaved to monomers by endogenous ribozymes and ligated probably by a yet unidentified host enzyme, yielding antigenomic circular monomers. These antigenomic molecules then serve as templates for genomic RNA using the same steps [14,15].

HDV does not encode its own replicase and there are no known HDV DNA intermediates. Accordingly, at least one host RNA polymerase (RNAP, traditionally thought to accept only DNA as templates) is involved in the replication and transcription of HDV. HDV replication and transcription takes place in the nucleus. HDAg mRNA is post-transcriptionally processed with a 5′-cap and a 3′-poly(A) tail, which are typical features of transcripts generated by RNAP II [16,17]. HDV RNA accumulation in cultured cells is sensitive to low doses of α-amanitin, an RNAP II inhibitor [18]. RNAP II associates with HDV RNA, both in infected cells and in vitro, and forms an active pre-initiation complex on HDV RNA, similar to what is observed on DNA promoters [19,20]. Additionally, because antigenomic RNA synthesis is more resistant to this transcriptional inhibitor, the involvement of at least one other host RNAP in HDV replication is proposed [21,22,23]. Consistent with this hypothesis, both RNAP I and RNAP III can associate with both polarities of HDV RNA and RNAP I colocalizes with HDV antigenomic RNA [23,24]. Although the role of RNAP II in both HDV replication and transcription is well established, there is still some controversy for the involvement of RNAP I and III.

## 3. Interaction with Host Cellular Proteins

Several host proteins involved in post-translational modifications, sub-cellular localization, RNA synthesis, and gene regulation, interact with HDAgs (reviewed by [25]). Among the proteins involved in post translational modification of the two HDAgs, the extracellular signal-related kinases 1 and 2 (ERK 1/2) phosphorylate HDAg-S at serine-177, and are necessary for the interaction of HDAg-S with RNAP II during antigenomic replication [26]. Another kinase, the casein kinase II (CKII) was shown to phosphorylate HDAg-S at the conserved Ser-2 position, a modification required for the activity of HDAg-S in HDV replication [27]. The double-stranded RNA-activated protein kinase R (PKR) was also shown to phosphorylate serine residues at positions 177 and 180, and a threonine at position 182 [28]. Several other proteins are also reported to post-translationally modify HDAgs, including protein farnesyltransferase (FTase), protein arginine methyltransferase 1 (PRMT1), p300 cellular acetyl transferase, small ubiquitin-related modifier isoform 1 (SUMO1), and Ubc9 [25].

HDAg-S was reported to co-immunoprecipitate with more than 100 host proteins and more than 25% of them were demonstrated to affect HDV RNA accumulation [29]. These proteins include RNAP II subunits, numerous kinases and helicases, transcription-related proteins, RNA binding and processing proteins, proteins involved in splicing, proteins implicated in chromatin formation and sub-cellular localization, and others involved in cell cycle division and apoptosis (Table 1). For example, HDAg-L directly interacts with the N-terminal domain of clathrin heavy chain (CHC) through its C-terminal domain at the trans-Golgi network, and this interaction was proposed to promote virion assembly [30]. 

Among the proteins reported to associate with HDV RNA genome, ADAR-1 interacts with HDV antigenome to edit the position corresponding to the stop codon of HDAg-S allowing the production of HDAg-L [12]. In addition to interacting with HDAgs, PKR also interacts with HDV RNA, and is proposed to protect viral RNA from nuclease digestion [31]. It is now well established that RNAP II has a crucial role in HDV genome replication and transcription, and in addition to its association with HDAgs, it interacts with the terminal stem-loop domains of both polarities of the HDV RNA genome [19]. Glyceraldehyde 3-phosphate dehydrogenase (GAPDH) also binds to HDV antigenomic RNA and increases cis-cleavage by enhancing HDV antigenomic ribozyme activity [32]. Recently, the main three paraspeckle proteins (PSF, p54nrb and PSP1), eEF1A1, hnRNP-L, and ASF were demonstrated to interact with HDV RNA [33,34,35].

## 4. How HDV Affects Its Host Cell

Because HDV interacts with numerous host proteins, several studies were initiated to understand the consequence of these interactions on gene and protein expression. The effect of the accumulation of HDAg and/or HDV RNA has been investigated using proteomic analyses in several experimental systems (Table 2). Using hepatocytes derived from the cellular carcinoma cell line (HuH-7) transfected with plasmids coding for HDV genomic RNA, antigenomic RNA or each HDAg individually, two studies reported several proteins differentially expressed [57,58,59,60]. These proteins are involved in pathways related to the regulation of nucleic acid and protein metabolism, transport, signal transduction, apoptosis and cellular growth regulation. Dysregulation was validated for triosephosphate isomerase (TPI), heat shock protein 105 (HSP105), heterogeneous nuclear ribonucleoprotein D (hnRNP D), histone H1-binding protein (NASP), La protein, and lamin C.

Another proteomic analysis reported a total of 89 proteins affected using a HEK-293 cell system mimicking HDV replication. This cellular system is comprised of three cell lines: 293 cells, 293 cells expressing HDAg-S under the control of a TET-ON promoter (293-Ag), and 293-Ag cells transfected with an HDV RNA genome unable to produce HDAg-S (293-HDV) [61]. In these cells, HDAg-S and HDV RNA altered the expression of 49 and 40 proteins, respectively. Differential expression of p53, ELAV, Transportin1 (TNPO1), Cofilin-1, Eukaryotic translation initiation factor 3 subunit D (EIF3D), and 10 kDa heat-shock protein (HSPE) were further validated. Moreover, the eukaryotic initiation factor 2 (EIF2) signaling pathway was one of the most affected pathways by HDV RNA accumulation with a total of 17 proteins differentially expressed. In this cell line, HDAg-S upregulates translational regulators and ribonucleotide binding activities, and disrupts pathways related to glycolysis/gluconeogenesis, pyruvate metabolism and cell cycle, more precisely the G2/M DNA damage checkpoint. These results are consistent with cell cycle disruption by HDV, but not with previous results showing a reduction of relative cell number in S and G2/M, with an increase in G1/G0 phase upon induction of HDV replication in this cellular system [62].

Active HDV replication induces the production of interferon beta (IFN-β) and interferon lambda (ifn-λ1/2/3) [63]. The pattern recognition receptor MDA5 was identified as the key component in HDV recognition. Found in the cytosolic compartment, MDA5 was proposed to detect HDV during HDV RNP export to form new virions. This receptor detects long dsRNA and higher-ordered RNA structures, and therefore HDV rod-shaped secondary structure could be important for MDA5 recognition. Although interferon (IFN) production rises upon HDV replication, it does not lead to HDV clearance. The innate immune response is widely reduced after 7 days of infection but only a moderate effect is observed on HDV replication. Thus, even after HDV-mediated innate immune response through MDA5 sensing, the interferon response caused only a limited inhibition of less than 50% of HDV replication, and this only at an early infection stage, showing that IFN therapy is not significantly effective to counteract HDV infection.

Recently, the involvement of paraspeckle proteins in HDV replication was demonstrated [33]. Paraspeckles are nuclear bodies found in proximity of nuclear speckles. They are still not fully understood, but can be induced by stress conditions and can sequester different RNA-processing/regulatory proteins [64,65]. Three major components of the paraspeckles interact with HDV RNA: the polypyrimidine tract-binding protein-associated splicing factor (PSF), the 54 kDa nuclear RNA-binding protein (p54nrb) and the paraspeckle protein 1 (PSP1) [33,34,56]. Knockdown of these proteins independently caused a reduction of more than 90% of HDV RNA abundance, indicating their importance for HDV RNA accumulation. Interestingly, upon HDV replication, PSP1 was shown to delocalize from the nucleus to the cytoplasm, and to co-localize with polyadenylate binding protein (PABP), a stress granule marker [33]. These results suggest that HDV induces a cellular stress response, and are consistent with the observed arrest in cellular cycle at the G1/G0 phase upon induction of HDV replication [62].

Also consistent with the cellular stress response induced by HDV, HDAg-L can activate the nuclear factor kappa B (NF-κB), as well as the signal transducer and activator of transcription-3 (STAT-3) [66,67]. Reactive oxygen species (ROS) production was increased and suspected to be induced through HDAg-L activation of oxidative stress pathway via NADPH oxidase-4 [67]. Excessive ROS production was previously linked with HBV replication and protein synthesis outcome [68]. Activation of these pathways could also be involved in the production of stress foci associated with HDV replication and could be linked with HDV liver pathogenesis [33]. In addition to these findings, morphological changes were observed upon HDV replication. Cells became rounder and smaller, a phenomenon that can be observed at the early apoptotic stage [33,62]. Many details are still missing on what precisely triggers stress foci formation in HDV replicating cells.

## 5. Challenges and Conclusions

Over the years, our knowledge of HDV, its replication, propagation and effects on the host has expanded. However, a lot remains unknown and more research is required to improve our understanding of HDV pathogenesis. Predominantly, most of what was reported on the contribution and disruption of host processes during HDV replication was obtained in vitro, or in conditions where HDAgs accumulation or HDV replication was triggered artificially in non-hepatocytes cells and in the absence of HBV. Although the restricted tissue tropism of HDV could be due to HBV, it is likely that the levels of HDAgs and HDV RNA are different during an infection, and together with the presence of HBV, might affect both interactions and host gene disruption. Because HDV is the only mammalian virus of its kind, but shares a lot of similarity with plant viroids, this virus might constitute a good model to study other subviral RNA pathogens, such as viroids [70,71]. Both HDV and viroids have a lot in common, including their similar genome structure, their self-cleaving ribozyme motifs, their replication method using a rolling-circle mechanism, which includes the redirection of host polymerases for their replication and transcription, and their high dependence on host factors to achieve their viral life cycle [72]. However, a major distinction is the requirement on HDAg-S for HDV replication. Although HDAg-S has been proposed to stimulate transcription elongation by RNAP II on HDV RNA by displacing the negative elongation factor (NELF) [49], its precise mechanism of action remains to be investigated and it is unknown if a similar activity is required for viroid replication in plants. Until now, several models have been developed to study HDV, to understand its replication, molecular biology and relationship with host cells and ultimately to better understand how the host responds to HDV infection. Understanding HDV interaction with host proteins and its repercussion on host cell will allow a better comprehension of its pathology and might reveal targets for the development of new treatments. Further research on HDV and viroids will lead to a better understanding of these viruses and how they disrupt host processes, and will eventually advance our comprehension of cell biology.

## Figures and Tables

**Table 1:** Host protein interacting with HDV RNA and/or HDAgs.

Host Protein (Cell Types Used)	Function	Interaction	Reference
Double-stranded RNA-activated protein kinase R (PKR) (HepG2, HeLa)	Phosphorylation (S117, S180, T182)	HDAg-S, RNA	[28]
Casein Kinase II (CKII) (HuH-7)	Phosphorylation (S2, S213)	HDAg-S	[27]
Protein Kinase C (PKC) (HuH-7)	Phosphorylation (S210)	HDAg-L	[27]
Extracellular signal-related kinases 1 and 2 (ERK1/2) (HEK-293T)	Phosphorylation (S177)	HDAg-S	[26]
Protein farnesyltransferase (FTase) (Cos-7, d H189, HuH-7, NIH3T3)	Isoprenylation with farnesyl (C211)	HDAg-L	[36,37,38,39]
Protein arginine methyltransferase 1 (PRMT1) (HuH-7)	Methylation (R13)	HDAgs	[40]
P300 cellular acetyltransferase (HeLa, HuH-7, and HepG2)	Acetylation (K72)	HDAgs	[41,42]
Small ubiquitin-related modifier	Sumoylation of lysine residues	HDAg-S	[43]
Isoform 1 (SUMO1) (HuH-7)			
Ubc9 (HuH-7)	Sumoylation of lysine residues	HDAg-S	[43]
Karyopherin (importin) 2α (BRL)	Nuclear import	HDAg-S	[44]
Nuclear export signal-interacting protein (NESI) (HuH-7, HepG2, COS7)	Nuclear import	HDAg-L	[45,46]
Lamin A/C (HuH-7)	Nuclear stability, chromatin structure and gene expression	HDAg-L	[45]
Clathrin heavy chain (HepG2, COS7, HuH-7)	Exocytosis	HDAg-L	[30,47]
Nucleophosmin (B23) (HuH-7 HepG2)	Nucleolar localization, shuttling, RNA synthesis/accumulation	HDAgs	[48]
DRB sensitivity-inducing factor (DSIF) (HeLa)	Relieves transcriptional repression; stimulates elongation by RNAP II	HDAgs	[49]
Delta interacting protein A (HEK-293)	Transcriptional regulation	HDAgs	[50]
Yin Yang 1 (YY1) (HeLa, HuH-7, HepG2)	RNA synthesis/accumulation	HDAgs	[41]
Histone H1e (COS7, HuH-7)	RNA synthesis/accumulation	HDAg-S	[51]
MOV10 (HuH-7, HEK-293)	RNA remodeling	HDAgs	[52]
Smad3 (HuH-7, Cos7)	Host gene expression	HDAgs	[53]
c-Jun (HuH-7, Cos7)	Host gene expression	HDAgs	[53]
TRAF2 (HEK-293, HuH-7)	Host gene expression	HDAgs	[54]
ZNF326 (HEK-293)	Transcription elongation	HDAg-S	[29]
CCAR1(HEK-293)	Helicase	HDAg-S	[29]
CDC5L (HEK-293)	Helicase	HDAg-S	[29]
Chromodomain helicase-DNA-binding protein 4 (CHD4) (HEK-293)	Remodeling of chromatin	HDAg-S	[29]
Centrosome-associated protein 350 (CEP350) (HEK-293)	Microtubule-organization at the centrosome	HDAg-S	[29]
Centrosomal protein 170kDa isoform alpha (HEK-293)	Microtubule organization	HDAg-S	[29]
H2A and H4 Histones (HEK-293)	Histone components	HDAg-S	[29]
Probable G-protein coupled receptor 179 precursor (HEK-293)	Signal transduction	HDAg-S	[29]
SC35 (HuH-7)	Splicing factor	HDAg-S, gRNA	[55]
Adenosine deaminase acting on RNA (ADAR 1) (HuH-7, HEK-293)	Post-transcriptional modification of HDV antigenome	agRNA	[12]
Glyceraldehydes 3-phosphate dehydrogenase (GAPDH) (HeLa)	Enhances delta ribozyme activity	agRNA	[32,34]
RNAP I (HeLa)	Antigenome synthesis	RNA	[24]
RNAP II (HeLa)	Genome synthesis, mRNA synthesis, Antigenome synthesis	HDAg-S, RNA	[19,20]
RNAP III (HeLa)		RNA	[24]
Polypyrimidine tract-binding protein associated splicing factor (PSF) (HEK-293, HuH-7)	Nuclear processes	RNA	[33,56]
54 kDa nuclear RNA-binding protein (p54nrb) (HEK-293, HeLa)	Nuclear processes	RNA	[33,34]
Paraspeckle protein 1 (PSP1) (HEK-293)		RNA	[33]
Heterogeneous nuclear ribonucleoprotein L (hnRNPL) (HeLa)	mRNA processing	RNA	[34]
Arginine/serine-rich splicing factor (ASF) (HeLa, HEK-293)	Splicing	RNA	[34,35]
Eukaryotic elongation factor 1A1 (eEF1A1) (HeLa)	Ribosomal aa-tRNA transport, gene expression	RNA	[34]

**Table 2:** List of host protein differentially expressed in the presence of HDV RNA and/or HDAgs.

Host Protein (Cell Types Used)	Biological Function	Reference
P53 (HEK-293)	Tumor suppressor and the regulation of cell cycle	[61]
Heat shock 10 kDa protein (HSPE) (HEK-293)	Chaperone, efficient protein folding	[61]
ELAV-like protein 1(HEK-293)	c-myc stabilization	[61]
Transportin 1 (HEK-293)	Receptor for nuclear localization signals	[61]
Eukaryotic Translation Initiation Factor 3 Subunit D (EIF3D) (HEK-293)	Translation initiation factor activity	[61]
Cofilin 1 (HEK-293)	ILK signaling pathway	[61]
14-3-3 σ (HEK-293)	Signal transduction	[61]
FAM136A (HEK-293)	Nuclear-encoded mitochondrial gene	[61]
BRI3BP (HEK-293)	Tumorigenesis, p53/TP53 stabilization	[61]
Histone H1 binding protein (NASP) (HEK-293)	Signal transduction; Cell communication	[29]
Triose phosphate isomerase (TPI) (HEK-293)	Metabolism; Energy pathways	[29]
Polyadenylate binding protein (PABP) (HEK-293)	RNA metabolism	[29]
Rho GDP dissociation inhibitor (GDI) (HEK-293)	GTPase activator	[29]
Guanine nucleotide-binding protein (HEK-293)	Signal transduction pathway	[29]
Brebrin 1 (HEK-293)	Cell growth and/or maintenance	[29]
Keratine 8 (HEK-293)	Cell growth and/or maintenance	[29]
Vinculin (HEK-293)	Cell growth and/or maintenance	[29]
Lamin C (HEK-293)	Cell growth and/or maintenance	[29]
Acetyl-CoA acetyltransferase (HEK-293)	Metabolism; Energy pathways	[29]
Zinc finger protein 326 (HEK-293)	Regulation of nucleobase, nucleoside, nucleotide and nucleic acid metabolism	[29]
High mobility group box 1 (HEK-293)	Regulation of nucleobase, nucleoside, nucleotide and nucleic acid metabolism	[29]
Guanine nucleotide binding protein (HEK-293)	Signal transduction; Cell communication	[29]
Serum albumin (HEK-293)	Transport	[29]
Heterogeneous nuclear ribonuclearprotein D (hnRNP D) (HuH-7)	mRNA metabolism and transport	[57]
Heat shock protein 105 (HSP105) (HuH-7)	Prevents the aggregation of misfolded proteins	[57]
Annexin IV (HuH-7)	Regulation of early stages of apoptosis	[57]
Proteasome activator (HuH-7)	Metabolism; energy pathways	[57]
NADH2 dehydrogenase (ubiquinone) flavoprotein 1 precursor (HuH-7)	Metabolism; energy pathways	[57]
Adenylate kinase 2B (HuH-7)	Metabolism; energy pathways	[57]
Eukaryotic translation initiation factor 2 subunit 1 (HuH-7)	Protein metabolism	[57]
Serine (or cysteine) proteinase inhibitor (HuH-7)	Protein metabolism	[57]
Heat shock 60 kDa protein	Protein metabolism	[57]
CKAP4 protein (HuH-7)	Cell growth and/ or maintenance	[57]
Tubulin alpha 6 (HuH-7)	Cell growth and/ or maintenance	[57]
Keratin 8 & Keratin, type I cytoskeletal 19 (HuH-7)	Cell growth and/ or maintenance	[57]
Dihydropyrimidinase related	Neurogenesis	[57]
Protein 2 (HuH-7)		
TRIM 28 protein (HuH-7)	Regulation of nucleobase, nucleoside, nucleotide and nucleic acid metabolism	[57]
DNA structure specific endonuclease FEN1 (HuH-7)	Regulation of nucleobase, nucleoside, nucleotide and nucleic acid metabolism	[57]
Ribonuclearprotein La (HuH-7)	Regulation of nucleobase, nucleoside, nucleotide and nucleic acid metabolism	[57]
High density lipoprotein binding protein (vigilin) (HEK-293, HuH-7)	Transport	[29,57]
N-ethylmaleimide-sensitive factor attachment protein (HEK-293, HuH-7)	Transport	[29,57]
Sorting nexin 5 (HEK-293, HuH-7)	Transport	[29,57]
Dopamine receptor interacting protein 4 (HuH-7)	Apoptosis	[57]
Interferon β/λ (HepG2, HuH-7, HepaRG)	Signaling proteins	[63]
Interleukine 8 (IL8) (HEK-293)	Antiviral protein	[33]
Nuclear Enriched Associated Transcript 1 (Neat1) (HEK-293)	Scaffold protein	[33]
Clusterin (HuH-7)	Role in tumorigenesis	[69]
